# Auditory and Language Abilities in Children with Takenouchi–Kosaki Syndrome: A Systematic Review

**DOI:** 10.3390/genes15080974

**Published:** 2024-07-24

**Authors:** Valeria Caragli, Elisabetta Genovese, Sara Parretta, Michele Pellegrino, Andrea Ciorba

**Affiliations:** 1 Otorhinolaryngology-Head and Neck Surgery, Audiology Program, University of Modena and Reggio Emilia, 41125 Modena, Italy; 2 Audiology Program, Department of Maternal, Child and Adult Medical and Surgical Sciences, University of Modena and Reggio Emilia, 41100 Modena, Italy; 3 ENT & Audiology Unit, Department of Neurosciences, University Hospital of Ferrara, 44121 Ferrara, Italy; andrea.ciorba@unife.it

**Keywords:** children, hearing loss, language, Takenouchi–Kosaki syndrome

## Abstract

Takenouchi–Kosaki syndrome (TKS) is a rare congenital disease caused by a de novo mutation in the Cell Division Cycle 42 (CDC42) gene. Patients with TKS present facial and body dysmorphisms, hematologic and immune dysregulation, intellectual disability, neurodevelopmental delay and hearing loss. The aim of this study is to review the literature, focusing on hearing and language abilities in children with TKS. A systematic search on PubMed, Scopus and Web of Science databases was performed, including twelve studies for a total of 13 patients. Hearing loss (HL) occurs in a great percentage of patients (84.6%); nonetheless, auditory threshold, severity of HL and language abilities were reported in a few cases. In two studies, auditory rehabilitation strategies were described. Although several studies have investigated the hematological features of TKS, still only a few authors have focused on the audiological and language abilities of these children. Given the fact that HL has a significant impact on behaviors, communications skills, and quality of life, it is important to adequately assess and rehabilitate patients early with this syndrome. Further studies are needed to improve the knowledge about this topic and improve the quality of life of patients with TKS.

## 1. Introduction

The Takenouchi–Kosaki syndrome (TKS) is rare a congenital disease caused by a de novo heterozygous mutation in the CDC42 (Cell Division Cycle 42) gene. In the majority of cases, patients present a p.Tyr64Cys mutation [1]; this gene variant is associated with the most severe form of the disease. CDC42 encodes a member of the RAS superfamily of low-molecular-weight GTP/GDP-binding proteins. In particular, it is involved in the intracellular signaling and biological processes of hematologic cells [2] and plays a major role in the control of a broad spectrum of activities, including hematopoiesis, neurodevelopment and hearing function [3].

In 2015, Takenouchi reported the first patient with TKS [4]; up to now, only a few other cases have been described. Patients with TKS present facial and body dysmorphisms and immunohematological dysregulations, including macrothrombocytopenia, lymphopenia, hypoimmunoglobulin, immunodeficiency, anemia and leukopenia. These conditions often require the administration of long-term corticosteroid therapy. Moreover, intellectual disability, neurodevelopmental delay and hearing loss have been reported—with a different degree of disease severity—in these patients [1,2,3,4,5,6,7,8,9,10,11,12,13,14].

Several studies have investigated the association between CDC42 mutations and hematologic disorders, focusing on pharmacological treatment approaches. Conversely, auditory function and language development in children with TKS has not been extensively analyzed so far. Consequently, there is not only a lack of knowledge on this topic but also on the possible rehabilitative strategies, on the communications outcomes and on the quality-of-life level of children with TKS [1,2,3,4,5,6,7,8,9,10,11,12,13,14].

The purpose of this study is to review the literature to identify the most common clinical features of patients with TKS, focusing on auditory and language abilities. The secondary aim is to present the most common therapeutic and rehabilitative strategies and the communication outcomes of these patients.

## 2. Materials and Methods

A literature search was performed through PubMed, Scopus and Web of Science databases according to the Preferred Reporting Items for Systematic reviews and Meta-Analysis (PRISMA) 2020 Statement guidelines [15].

The selection of articles was performed by 3 authors. After obtaining records from the databases, one author reviewed abstracts and titles and excluded duplicated records and articles not directly related to TKS. After this first screening procedure, all the full texts of the remaining studies were read by all the authors independently, filling a database with important notes and/or reasons for exclusion. Finally, each author reviewed the two other databases, and all the relevant data were included in a single table (Table 1).

**Figure 1:**
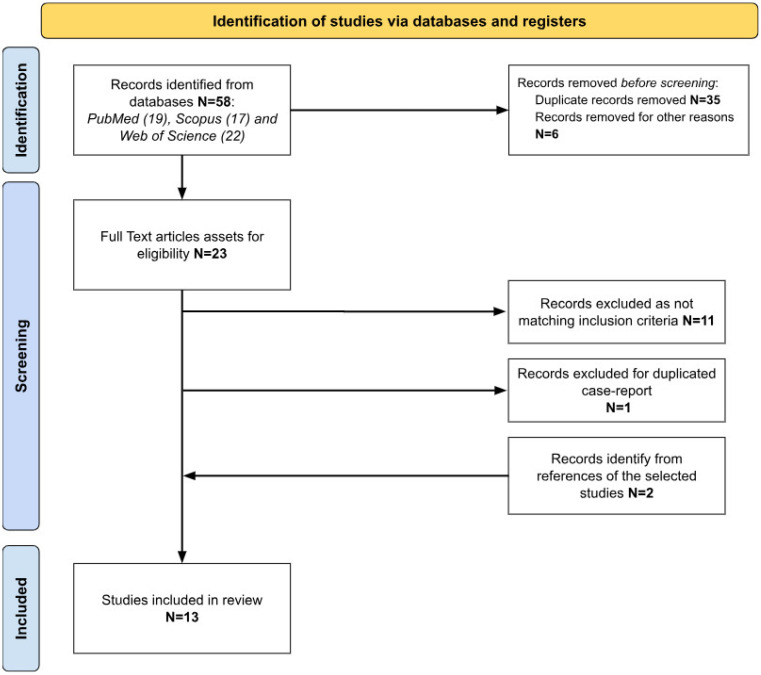
Identification process of the selected studies according to PRISMA criteria.

The search was performed without any filter, including range, time and using the term “Takenouchi Kosaki syndrome”. Last search was performed on 14 February 2024. Eligibility of the articles was assessed using two main inclusion criteria: (1) studies including patients with genetically confirmed TKS; (2) studies with clinical description of cases. All texts examined were written in English or French—languages spoken by the authors of this review. Articles without a report of clinical phenotype or without genetic analysis confirming TKS were excluded. Patients’ data, extracted from the selected studies, included gender, age, genetic analysis, clinical features, laboratory data, imaging, audiology and language skills, therapy and rehabilitation approaches. Figure 1 summarizes the identification process of the selected articles.

Data were analyzed in accordance with the Helsinki Declaration, the Italian privacy and sensitive data laws, and the in-house regulations of our hospital.

## 3. Results

A total of 58 studies were retrieved, 13 of which were included in the present study (Figure 1). Table 1 summarizes the data of the included reports.

### 3.1. Population

Patients’ population is composed of 13 subjects; of these, 69.2% were female and 30.8% were male. The mean age was 13.5 years (0.75–26).

Studies were conducted in different countries (Table 1).

### 3.2. Genetic Analysis

Genetic analysis identified a de novo heterozygous mutation in the CDC42 gene in all patients. The most common variation was a de novo c.191A>G p.Tyr64Cys missense mutation. It was carried by nine patients (69.2% of the cases). In the other percentage of cases, patients presented a different variation of the mutation, such as p.Y64C (7.7%), c.203G>A p. Arg68Gln (7.7%), c.68A>G p.Tyr23Cys (7.7%) and c.242G>A p.Cys81Tyr (7.7%).

### 3.3. Clinical and Laboratory Features

Physical and facial dysmorphisms were the most common features reported and described in these patients (100%). These conditions were almost always associated with thrombocytopenia (92.3%), a developmental delay (84.6%) and intellectual disabilities (IDs) (46.1%). The disease was comorbid in 76.9% of cases, with immunological alterations such as hypogammaglobulinemia (38.4%), leukocytopenia (30.8%), anemia (23%) and lymphopenia (15.3%).

### 3.4. Imaging

Neuroimaging was performed with MRI (69.2%) or CT scans (7.7%). Ventriculomegaly was present in 84.6% of patients who underwent MRI, corpus callosum hypoplasia in the 15.4% of cases and cerebellar atrophy in the 38.5% of cases. Two different radiological variants, the Dandy–Walker variant [5] and hypoplastic cerebellar vermis, were reported [6]. Normal findings were reported in another case [7].

### 3.5. Audiological Evaluation

The audiological evaluation was reported in 11 out of 13 studies. In the study where it was not reported, a hearing test was recommended. In 10 (76.9%) patients, sensorineural hearing loss (SNHL) was diagnosed. The extent of hearing loss severity ranged from mild to profound [2,6,8]. One patient was diagnosed with “deafness” [1], and no further data or information were available. Two cases reported on an audiological assessment through an auditory brainstem response [2,8].

### 3.6. Therapy and Rehabilitation

Auditory rehabilitation with hearing aids (HAs) was proposed in a subject. In this case, the patient with the aided threshold was able to understand simple requests and developed language skills; however, they were limited due to his severe ID [4]. In the presence of mild hearing loss and ID, specific programs at schools and individualized follow-up cares were proposed [9].

One patient, who was not able to read nor write, used an iPad with a Pro Logic II program for general communication, primarily at school and at home, due to anxiety in other settings [2].

For recurrent ear infections, two studies described the use of myringotomy and tympanostomy tubes [3,12].

None of the identified subjects were treated by other means, such as a cochlear implant.

## 4. Discussion

Takenouchi–Kosaki syndrome is a rare genetic disease caused by a mutation in the CDC42 gene. The syndrome is characterized by the presence of hematologic disorders and body abnormalities, which are often in comorbidity with neurophysiopathological impairments and hearing loss of a different entity [7,9]. Although several variants were described, the p.Tyr64Cys mutation was detected in the vast majority of cases (69.2%). The derived mutant allele determined a hypomorphic effect of the gene and caused a reduction in GTPase activity. GTPase is a RHO family protein that regulates a wide range of functions, such as cell morphology, cell migration, cell cycle and actin dynamics [16,17]. The modified functioning of GTPase activity explains the multiple symptoms of TKS. In particular, the presence of the p.Tyr64Cys mutation seems to be responsible for the most prototypic and severe forms of the disease [2,5,7,9,10,11,18,19,20].

As proposed by Hamada et al. (2020), the p.Tyr64Cys mutation affects Cdc42-dependent cell polarity organization, and it is critical for axon elongation, dendritic arbor formation and migration during the corticogenesis; these processes could underlie the pathophysiology of ID [16,17,21] and, eventually, sensorineural hearing loss.

Despite there being a significant number and a high prevalence of patients with TKS and audiological impairment (84.6%), only a few studies have focused on the description of the hearing threshold and levels of language development over the recent years, as well as on the different possible auditory rehabilitation strategies [1,2,3,4,5,6,7,8,9,10,11,12,13,14]. Consequently, there are no significant data to discuss and compare on this topic. According to the results of the present review, HL may occur at different degrees of severity, ranging from mild to severe. According to the findings within the literature, HL severity seems to be strongly related to the variant type of the mutated gene. In particular, the presence of the p.Tyr64Cys mutation could be related to a more severe degree of HL.

It is well known that HL has a negative impact on the development of language and speech abilities, on behavioral disorders and on the quality of life of people with it [22]. Consequently, it is very important to assess patients’ hearing threshold early and to adequately rehabilitate their auditory functions. Thus, common rehabilitation strategies include hearing aids, alone or in combination with augmentative alternative communication (AAC) tools [9,11]. Nonetheless, good results may be obtained by also using hearing aids, cochlear implants and tactile vibrators devices. Children with TKS may also benefit from speech and language therapy in order to improve both perceptive and receptive abilities.

Interestingly, five studies were from Japan, two from Poland, two from USA, one from Italy, one from South Africa, one from Canada and one from Belgium.

Due to the complexity of the TKS condition, a multidisciplinary assessment of these patients is recommended. Therefore, an audiological and phoniatric evaluation is mandatory to detect the level of these patients’ auditory and language skills and propose adequate rehabilitation strategies to improve their communication skills and quality of life. The auditory strategies available, at present, may not be fully effective in rehabilitating these subjects; hopefully, further approaches will be eventually identified and proposed in the future.

It is likely that a valid solution for patients to fully recover from TKS will be gene therapy [23]. In this regard, recent studies suggested that genetic-based nanotechnological strategies are effective for treating different diseases, including HL [24]. However, up to now, the limited number of pathological samples as well as the inaccuracy of immortalized cell lines and mouse models used hamper the establishment of ideal models for rare diseases. These obstacles may be overcome by using induced pluripotent stem cell (iPSC)-based models. Disease-specific iPSCs have been used to understand cell functions. To date, studies on TKS patient-derived iPSCs (TKSiPSCs) demonstrated to be the more effective cellular model for investigating macrothrombocytopenia in a stepwise procedure throughout the differentiation process from pluripotent stem cells (PSCs) to platelets [10].

It is possible to speculate that future studies using iPSCs will also eventually be able explain the occurrence of hearing impairment in TKS and may potentially be used to recover hearing loss in these patients [10]. Nonetheless, all these approaches should be reconsidered and eventually tailored according to patients’ global medical conditions and their specific needs. As children with TKS may improve auditory and develop language skills, even if at a lower rate compared to their counterparts without a hearing impairment, adequate follow-ups should always be performed.

Drawbacks: the main limitation of this study is the limited number of studies on TKS that are available, with there being insufficient data on auditory function and language levels regarding the described cases.

## 5. Conclusions

The present review shows that hearing loss is a common feature of patients with TKS, a rare congenital syndrome presenting body and facial dysmorphism and hematological alterations due to a mutation of the CDC42 gene, which encodes an RHO family GTPase.

Different variants of the mutation [the main one is c.1449T > C/p. (Tyr64Cys)] describe different phenotypes and severity of comorbidities, which can include intellectual disability, development delay, lymphedema, hypothyroidism, recurrent infections and sensorineural hearing loss.

Audiological testing and eventual related rehabilitative interventions should always be performed in the case of a TKS diagnosis. In fact, assessing auditory thresholds and language abilities is crucial to provide these children with the opportunity of developing appropriate communication skills and achieving an adequate quality of life.

There is still insufficient information available on this topic, and further studies are necessary, particularly involving the molecular pathophysiology of this disorder. Hopefully, in the future, genetic therapeutic approaches will be developed for this condition, as per other similar conditions.

## Figures and Tables

**Table 1:** Descriptive data of patients.

Reference	Patients	Country	Genetic Analysis	Clinical Features	Laboratory Data	Imaging	Audiology/Language	Therapy/Rehabilitation
Yamano et al., 2022 [1]	1 F 15 y	Japan	CDC42, c.191A>G p.Tyr64Cys NM_001791.4	Facial and body dysmorphism, severe DD	Macrothrombocytopenia, hypogammaglobulinemia, anemia	CT: enlarged spleen	Deafness	Corticosteroids, rituximab, transfusions, splenectomy
Martinelli et al., 2018 [2]	1 F 15 y	Canada	CDC42, c.191A>G p.Tyr64Cys NM_001791.3	Facial and body dysmorphism, DD, ID, recurrent airway infections	Macrothrombocytopenia, leukocytopenia, hypogammaglobulinemia, hypothyroidism	MRI: ventriculomegaly, thin corpus callosum, white matter mildly thin. RX: subtle scoliosis and kyphosis.	Profound SNHL on right side; significant SNHL on the left side (ABR)	She does not write or read, but used an iPad with a Pro Logic II
Szczawinska-Poplonyk et al., 2020 [3]	1 M 11 y	Poland	CDC42, c.242G>A p.Cys81Tyr NM_044472.3	Facial and body dysmorphism, neuro-DD	Thrombocytopenia, leukocytopenia, anemia (pancytopenia), hypogammaglobulinemia	MRI: IV stage nodular sclerosis (NS) Hodgkin’s lymphoma CT chest: fibrosis, bronchiectasis, nodules CT abdomen: enlarged spleen, hypoplastic left kidney, lymphadenopathy forming a mass 8.5 cm long	SNHL, recurrent ear infections	Antibiotics, antiviral and antimycotic, corticosteroids, transfusions, chemotherapy. Tympanostomy tube and drainage of maxillary sinuses.
Takenouchi et al., 2015 [4]	1 F 22 y	Japan	CDC42, c.191A>G p.Tyr64Cys NM_001039802.1	Facial and body dysmorphism, DD, ID, lymphedema	Macrothrombocytopenia, leukocytopenia	MRI: ventriculomegaly, cerebellar atrophy	SNHL	NR
Bucciol et al., 2020 [5]	1 F 26 y	Belgium	CDC42, c.191A>G p.Tyr64Cys	Facial and body dysmorphism, immunodeficiency, DD, growth retardation, feeding disease, recurrent airway infections	Lymphopenia, neutropenia, anemia, thrombocytopenia	MRI: Dandy–Walker variant (cerebellar atrophy), ventriculomegaly	SNHL	NR
Szczawińska-Popłonyk et al., 2023 [6]	1 M 9 m	Poland	CDC42, c.191A>G p.Tyr64Cys NM_001791.4	Facial and body dysmorphism	Macrothrombocytopenia, hypogammaglobulinemia, lymphopenia	MRI: hypoplastic cerebellar vermis (cerebellar atrophy), ventriculomegaly	Bilateral-profound SNHL	Antibiotic prophylaxis
Flynn et al., 2021 [7]	1 F 10 y	South Africa	CDC42, c.68A>G p.Tyr23Cys NM_001791.4	Facial and body dysmorphism, neuro-DD, ID, behavioral disease	NR	CT brain scan: normal	NR	NR
Santoro et al., 2021 [8]	1 F 10 y	Italy	CDC42, c.191A>G p.Tyr64Cys NM_001791.4	Facial and body dysmorphism, DD, ID, lymphedema	Macrothrombocytopenia	MRI: ventriculomegaly	Mild bilateral SNHL 15-20 dB (ABR)	No need of hearing aids
Motokawa et al., 2017 [9]	1 F 12 y	Japan	CDC42, c.191A > G p.Tyr64Cys NM_001039802.1	Facial and body dysmorphism, severe motor DD, severe ID, recurrent airway infections	Macrothrombocytopenia, hypogammaglobulinemia, hypothyroidism	MRI: corpus callosum hypoplasia, ventriculomegaly	SNHL	Special-needs school
Uehara et al., 2019 [10]	1 M 4 y	Japan	CDC42, c.191A>G p.Tyr64Cys	Facial and body dysmorphism, lymphedema, DD	Macrothrombocytopenia, hypothyroidism	MRI: cerebellar atrophy, ventriculomegaly	SNHL	NR
Takenouchi et al., 2016 [11]	1 F 18 y	Japan	CDC42, c.191A>G p.Tyr64Cys NM_001039802.1	Facial and body dysmorphism, DD, ID, ataxia, lymphedema, recurrent airway infections	Macrothrombocytopenia, leukocytopenia	MRI: cerebellar atrophy, ventriculomegaly	SNHL	Hearing aids
McAuley et al., 2022 [12]	1 F 15 y	USA	CDC42, p.Y64C	Facial and body dysmorphism, unilateral lymphedema, DD	Macrothrombocytopenia, hypogammaglobulinemia, lymphopenia	NR	NR, recurrent ear infections	Tympanostomy tube
Stubbs et al., 2021 [13]	1 M 17 y	USA	CDC42, c.203G>A p. Arg68Gln	Facial and body dysmorphism, hydrocephalus, OSAS, congenital lobar emphysema	Macrothrombocytopenia	NR	Bilateral SNHL	Corticosteroids

DD: developmental delay, F: female, ID: intellectual disability, M: male, NR: not reported, SNHL: sensorineural hearing loss.

## Data Availability

The authors declare that the data supporting the findings of this study are available within the article.
